# Preliminary study of physicochemical, thermal, rheological, and interfacial properties of quinoa oil

**DOI:** 10.12688/f1000research.134134.1

**Published:** 2023-11-15

**Authors:** Cristhian Camilo Castaño-Ángel, Jesús Alexander Tarapues-Cuasapud, Jesús Eduardo Bravo-Gómez, Jose Fernando Solanilla-Duque, Diego Fernando Roa-Acosta

**Affiliations:** 1 Departamento de Agroindustria, Facultad de Ciencias Agrarias, Universidad del Cauca, Comuna 1, Cauca, 190001, Colombia

**Keywords:** pseudocereal, antioxidant, oil extraction, interfacial properties, rheology

## Abstract

**Background:** The growing popularity of nutrient-rich foods, among which is quinoa, is due to the increasing demand for healthier choices. Oils and hydrolyzed proteins from these foods may help prevent various health issues. The objective of this work was to perform extraction from the endosperm of the grain from high-protein quinoa flour by physical means *via* a differential abrasive milling process and extracting the oil using an automatic auger extractor at 160°C, as well as characterizing extracted oil.

**Methods:** Quinoa oil extraction and physicochemical characterization were carried out. Chemical and physical quality indexes of quinoa oil were established, and both characterizations were conducted based on international and Columbian standards. Thermal properties were evaluated by differential scanning calorimetry, and rheological and interfacial properties of the oil were evaluated using hybrid rheometers and Drop Tensiometers, respectively, to determine its potential for obtaining functional foods.

**Results:** The result was 10.5 g of oil/ 100 g of endosperm, with a moisture content of 0.12%, insoluble impurities of 0.017%, peroxide index of 18.5 meq O _2_/kg of oil, saponification index of 189.6 mg potassium hydroxide/g of oil, refractive index of 1.401, and a density of 0.9179 g/cm ^3^ at 20°C. Regarding contaminating metals, it presented 7 mg of iron/kg of oil, a value higher than previously established limits of 5 mg of iron/kg of oil. The oil contained 24.9% oleic acid, 55.3% linoleic acid, and 4% linolenic acid, demonstrating antioxidant capacity. Quinoa oil showed thermal properties similar to other commercial oils.

**Conclusions:** The interfacial and rheological properties were suitable for the stabilization of emulsions, gels, and foams, which are important in various industrial applications and could facilitate the development of new products. The extracted quinoa oil presented similar characteristics to other commercial oils, which could make it a potential product for commercialization and application in different industries.

## 1. Introduction

Quinoa is a dicotyledonous plant of the Amaranthaceae family, herbaceous, annual, or biennial. Its morphology, coloration and phenology depend on the agroecological zones where it is grown. It has a high plasticity to adapt to different edaphic conditions, drought, frost and salinity. ^1^ The production of quinoa grain for consumption has been growing exponentially in the world. ^2^^–^^4^ During the 1980s, quinoa was cultivated both commercially and non-commercially in seven countries. However, at present, the cultivation of quinoa has expanded significantly, with over 95 countries now reported to be involved in its production. ^5^ Such a situation has been gaining importance in Colombia, where until a few years ago, research was limited by the lack of diversity in the genetic material present and cultivated within the country. ^6^

There are around 100 quinoa cultivars, whose grains are prepared in various ways for direct consumption and transformed into multiple derivatives. ^7^ In most cultivars, the endosperm is rich in fats and proteins, components that are stored in the lipid and protein bodies. ^8^ While, the perisperm contains uniform cells filled with starch granules. ^9^ These granules are present as individual entities or as spherical aggregates. ^10^ In order to achieve the separation of the anatomical parts of the grain, differential grinding processes have been implemented. ^11^^,^^12^ This type of process is completely dry, without dissolution or filtration stages, which does not produce liquid or polluting effluents. ^13^ By subjecting the grain to abrasive grinding, the content of protein, fat and ash increases in the separated endosperm compared to the whole grain, this process of separation of external grain tissues has been studied by other authors. ^14^^,^^15^

The oil content in the quinoa grain ranges from 1.8% to 9.5%, with a calculated global mean of 5.8%. ^16^ In the composition of lipids, unsaturated fatty acids dominate, highlighting its high content of linoleic acid (50.2-56.1%) and oleic acid (22.0-24.5%), and moderate of linolenic acid. ^17^ The presence of linoleic and oleic fatty acids normally makes oils susceptible to oxidative rancidity. In the case of quinoa oil, its tocopherol content provides antioxidant protection against oxidative rancidity. ^18^ The extraction of quinoa oil by means of supercritical carbon dioxide has shown good extraction performance while preserving the quality of the oil, ^19^ whereas the extraction of quinoa oil by the wet method (solid-liquid) and with ultrasound pretreatment are also effective. ^20^^,^^21^ Authors such as Mufari *et al*., ^22^ have reported oxidative stability and characterization of quinoa oil extracted from wholemeal flours and from the endosperm (germ), the results showed a greater extraction from the endosperm. Because the information on the extraction and characterization of quinoa oil is limited, the objective of this work is to carry out the extraction by physical means and characterize the oil extracted from the endosperm of the grain.

## 2. Methods

### 2.1 Quinoa grain

The quinoa grain ( *Chenopodium quinoa* Willd.), cultivar Nariño, supplied by SEGALCO SAS, was used. The germ was obtained by the abrasive milling process (GranEL, model C-100, Bogotá, Colombia), that had an abrasion chamber in order to obtain flours and polished grain from 1,000 kg of quinoa grains as described in Roa-Acosta *et al.* ^13^

### 2.2 Quinoa oil extraction and physicochemical characterization

A commercial type of automatic oil extractor machine (DULONG, Canton, China) was used, which has an alternating current motor of 450 W, handling a voltage of 110 volts, equipped with a worm screw and a thermal resistance, to perform a cold or hot extraction. It has a processing capacity of 2 kg/h. In total, 400 g of flour was recirculated from the germ to obtain a higher extraction. The resistance of the equipment was adjusted to 120°C. ^21^^,^^22^ To clean the oil, it was subjected to centrifugation at 5,000 rpm for 5 minutes. Subsequently, it was decanted in 120 mL amber bottles for 24 hours at a lower temperature of 15°C, to separate the fine particles that were not eliminated in the centrifugation.

2.2.1 *Density*

A 25 mL Gay-Lussac pycnometer was used for all density measurements. The volume of this pycnometer was calibrated between 4 and 45°C using deionized water. It was filled with oil to the limit avoiding the presence of bubbles when covering it with the stopper. Finally, it was weighed to the nearest 0.1 mg. The determination was carried out in triplicate. ^23^

2.2.2 *Moisture content and volatiles*

Oil (3 ± 0.001 g) was weighed and heated at 103°C for 1 h. Subsequently, the sample was cooled in a desiccator to room temperature and weighed at 1 hour intervals until a constant weight was achieved. The determination was carried out in triplicate. ^24^

2.2.3 *Insoluble impurities*

The oil was weighed (20 ± 0.01 g) and 150 mL of petroleum ether was added. Then, it was allowed to stand at 25°C for 30 minutes. The oil was filtered on a 589/3 grade paper, previously dried and weighed, with the help of a funnel and a vacuum pump. Finally, the filter paper was evaporated in a convection oven at 103°C for 1 hour and then weighed to the nearest 0.001 g. The determination was carried out in triplicate. ^24^

2.2.4 *Color properties*

The color of the samples was determined with a spectrophotometer (CM5 colorimeter, Konica Minolta, Tokyo, Japan). In this analysis, the color and appearance of transparent, opaque, or translucent samples were evaluated. The color attributes of the oil were measured using a spectrophotometer (CM5, Konica Minolta Inc., Tokyo, Japan) in reflectance mode. Standard black and clear quartz plates were used to calibrate the spectrophotometer. Samples were loaded into a 10 mm cuvette using standard D65 illumination as the light source and the standard observer setting of 10° (10° dihedral angle) was chosen. The results were expressed as CIELAB L*, a* and b* values by triplicate. The wavelength range was 360 to 740 nm. Calibration of the equipment was performed with a black plate provided by the manufacturer and zero and blank calibration was performed. In total, 10 mL of sample was taken in the quartz cell and the parameters L*, a* and b* were determined. Color was performed in triplicate. In color space, L* indicates lightness, a* and b* are the color directions: +a* is the red axis, -a* is the green axis, +b* is the yellow axis and -b* is the blue axis. Saturation was calculated with the equation described by Gutiérrez *et al*. ^25^ Saturation was calculated by using Equation 1. The color of sample was used as reference for the determination of the total color difference of each sample. ^25^ All measurements were performed in triplicate.

$$∆E^{*}=\sqrt{{\left(∆L^{*}\right)}^{2}+{\left(∆a\right)}^{2}+{\left(∆b^{*}\right)}^{2}}$$

### 2.3 Physical and chemical quality indexes

2.3.1 *Refractive index*

It was carried out according to the instructions of the Abbe Mark II refractometer equipment, on a scale of 1.3 to 1.7. After each measurement, the prism surface was cleaned with a soft cloth and a cotton swab moistened with hexane. The refractive index was determined at 20 and 25°C using Equation 6 (liquid oil at these temperatures). ^26^^–^^28^

### 2.4 Peroxide index

This index was determined according to the procedure described in AOAC standard 965.33. We mixed 5.0 g of oil in 25 mL of an acetic acid/chloroform solution (3:2), stirring until complete dissolution. Then, 0.5 mL of a saturated solution of potassium iodide was added and allowed to stand in the dark for 60 minutes. Subsequently, 75 mL of hot water was added and 0.5 mL of 1% starch solution was added, forming a dark color. This solution was titrated with 0.01 N sodium thiosulfate until the color disappeared. The determination was carried out in triplicate.

### 2.5 Free fatty acid index

This determination was carried out according to AOAC 940.28. In an Erlenmeyer, 7.05 ± 0.01 g of oil was weighed, 50 ml of ethanol (95% purity), 2 ml of phenolphthalein and 15 drops of 0.1M sodium hydroxide were added. This solution was titrated with NaOH 0.25 M, stirring constantly until a permanent faint pink appears and persists. The determination was carried out in triplicate. The acid number is expressed as a percentage of oleic acid (molecular weight 280.4472 g/mol).

### 2.6 Saponification index

The saponification index was determined according to AOAC 920.160. A total of 2.3 g ± 0.01 g of sample was weighed into an Erlenmeyer flask, 25 ml of 4% ethanolic potassium hydroxide was added and refluxed at 80°C for one hour. It was dismantled and 25 ml of ethanol (95% purity) and three drops of phenolphthalein 1% were added. This solution was titrated with 0.5N HCl until a yellow coloration was observed, using an automatic titrator (848 Titrino plus, Metrohm, Switzerland). The determination was carried out in triplicate.

### 2.7 Iodine index

This determination was carried out according to AOAC 920.159. In an Erlenmeyer flask lined with aluminum foil, 0.08 g of oil was weighed. Then, 8 mL of a solution of hexane and acetic acid was added at a ratio of 1:1, and after shaking, 10 mL of Wijs solution was added. The Erlenmeyer was capped and placed at room temperature in the dark for 30 minutes. After this time, 8 mL of KI 15% were added and little by little 40 mL of previously boiled and cold water was added. This solution was titrated with 0.1 N sodium thiosulfate until the yellow color disappeared. To this solution, 1 mL of 1% starch solution was added and titration was continued until the blue color disappeared. The determination was carried out in triplicate.

### 2.7 Metallic trace elements

The determination of traces of copper, iron and nickel in quinoa oil was carried out by the atomic absorption method. Previously, 20 μL of oil was diluted in petroleum ether in a 1:3 ratio and evaporated in a graphite furnace. The UV-Vis spectrometer (Genesys 10S, Thermo Scientific, USA) was calibrated with standard solutions of organic compounds of the metals tested. The wavelengths used for the determination of copper, iron and nickel were 327.7, 302.1 and 232 nm, respectively. For the determination of iron, a graphite tube coated with niobium on the inside was used.

### 2.8 Bioactive properties

2.8.1 *Lipid profile*

Lipid profiles were measured using a gas chromatograph (Agilent 7890A, Agilent Technologies, Switzerland), equipped with a flame ionization detector (FID), auto sampling and auto injection system (Agilent 7683B, Agilent Technologies, Switzerland) and OpenLab ChemStation software, version B.04.01 for data capture was used (open source alternative, OpenChrom). An HP-5MS UI fused silica capillary column (30 m × 0.25 mm ID, film thickness 0.25 μm, J&W. Scientific) was used. In the assay, operating conditions were as follows: 1 μL of sample was taken and subjected to a first heating ramp, the initial oven temperature was at 50°C for 5 min, then at 150°C at 4°C/min and held for 5 min with a slope of 5°C/min to a maximum temperature of 220°C at 4°C/min with a waiting time of 15 min. The second heating ramp for cleaning of any debris that may have remained in the column was carried out at a slope of 40°C/min with a maximum temperature of 250°C and a waiting time of 2 min. The injector and detector temperatures at 275 °C; injection volume, 0.2 μL; split ratio, 50:1. Helium was used as carrier gas and injected at 1 mL/min. ^29^

2.8.2 *Oil oxidative stability*

Addition of the synthetic antioxidant butylated hydroxytoluene (BHT) was performed at a concentration of 0.02%, described by Mousavi *et al*. ^30^ Samples of 3 g with and without addition of the antioxidant were taken and placed in the reaction cells in the Rancimat 743 apparatus (Metrohm, Herisau, Switzerland) at a temperature of 110°C and an air flow of 20 L/h. The induction time was obtained for quinoa oil with and without the addition of BHT.

### 2.9 Thermal properties

The melting and crystallization point was determined was studied by using differential scanning calorimetry (DSC) analysis,. 1-Butanol for presenting a low melting point of -89.8°C and a boiling point of 117.7°C used as a reference for the analysis. The sample size was 10 μL, with a temperature setting from -80 to 60°C, with a cooling and heating rate of 20°C/min and 10°C/min, respectively, with isotherms at the same for 1 min, repeated twice. ^15^

### 2.10 Rheological properties

The rheological properties of quinoa oil were measured using a rheometer (Discovery HR-3 rheometer, TA Instruments, USA) equipped with a parallel plate geometry (40 mm diameter). The quinoa oil was subjected to shear rate from 0.1 s ^-1^ to 100 s ^-1^ at 15°C, 25°C and 35°C. A strain sweep from 0.01% to 100% was performed to determine the region of linear viscoelasticity at a constant frequency of 0.1 Hz. Then, a frequency sweep was performed at a constant strain (0.1%) and in the range of 0.062 rad s ^-1^ to 62 rad s ^-1^. The quinoa oil was subjected to a heating and cooling cycle from 15°C to 65°C at 6.2 rad s ^-1^. We measured viscous and elastic modulus for different frequency and temperature sweeps. We applied a power model to analyze both flow and viscoelastic tests. ^31^

### 2.11 Interfacial properties and dilatational rheology

2.11.1 *Adsorption kinetics*

The interfacial tension of virgin quinoa oil obtained by mechanical extraction was measured using a drop tensiometer (Tracker H, Teclis instruments, Tassin, France) using the Rising method. ^32^^–^^34^ The sample was placed in a 10 mL quartz cuvette, and a 3 mm diameter stainless steel capillary tip was submerged inside to form an air bubble. All measurements were conducted at room temperature. The interfacial tension data measured as a function of time were analyzed using nonlinear numerical fitting expressed by the following equation ^31^^,^^35^:

$$y=y_{0}+A_{1}e^{-x/t_{1}}+A_{2}e^{-x/t_{2}}$$ (2)

where, *y*_0_ represents the equilibrium interfacial tension and the exponential terms (decay rate) correspond to the known adsorption kinetics (or adsorption constants) for proteins and other macromolecules. Therefore, the penetration mechanism ( *K*_d_) of the interface is described by the first exponential decay, while the reordering mechanism ( *K*_p_) of the molecules in the interface is described by the second exponential decay. The individual decay rates (penetration and ordering) were obtained using the relationships *K*_d_ = 1/t _1_ y *K*_p_ = 1/t _2_.

2.11.2 *Dilatational rheology and viscoelastic properties*

The dilatational rheology of the air/oil interface was analyzed by sinusoidally oscillating the volume of an air bubble in the Rising method setup. The method consisted of a periodic, automated, and sinusoidal compression and expansion of the interface, performed by decreasing and increasing the volume of the drop with deformation amplitudes of ∆A/A = 5 and 10% (the percentage change in area is in the linear viscoelastic range), with measurements taken at an angular frequency of 100 Hz. The sinusoidal oscillation for the measurement of the dilatational surface was performed with five ^5^ oscillation cycles followed by one ^1^ cycle of no oscillation. The average standard precision of the surface pressure was approximately 0.1 mNm ^−1^. This perturbation in the interface allows the adsorbed molecules to reduce the tension depending on the adsorption or desorption of the solution, giving rise to relaxation processes and the pseudo-thermodynamic equilibrium of the system. The surface rheological parameters *E*, *E _d_*, *E _v_*, and *ϕ* were measured as a function of the adsorption time ( *θ*). The interfacial dilatational modulus (E) derived from the change in interfacial tension (dilatational tension) corresponds to the resistance to surface perturbations *α* ( Equation 3), resulting from the small change in surface area (dilatational tension) A ( Equation 4) ^32^^,^^36^^,^^37^:

$$\sigma =\sigma_{0}\;\sin\;\left(\omega ∙\theta +f\right)$$ (3)

$$Α={Α}_{0}\;\sin\;\left(\omega ∙\theta \right)$$ (4)

$$E=\frac{\mathrm{d\sigma}}{\frac{\mathrm{dA}}{A}}=-\left(\frac{\mathrm{d\pi}}{\text{dlnA}}\right)=\left|E\right|e^{i}=E_{d}+iE_{v}$$ (5)

$$\tan\delta =\left(\frac{E_{v}}{E_{d}}\right)\;$$ (6)

Where σ _0_ and A _0_ are the amplitudes of stress and strain, respectively, q is time, f is the phase angle between stress and strain, w is the angular frequency, and | *E*|, is the absolute modulus, and the ratio (σ _0_/A _0_) is a measure of the total material unit of resistance to dilation deformation (elastic + viscous). ^32^ The interfacial dilation modulus ( *E*) is a complex quantity and composed of real and imaginary parts ( Equation 5). The real part ( *E*_*d*_) is called the storage modulus ( *G*′) and represents the elastic energy stored in the interface ( *i.e.*, the elasticity of dilation), *E*_*d*_ = | *E*|(cos). The imaginary part ( *E*_*v*_) is called the loss modulus ( *G*″) and represents the dissipation of energy in the relaxation process ( *i.e.*, the viscosity of dilation), *E*_*v*_ = | *E*|(sin), and f is the phase angle between stress and strain, that is, a measure of the relative elasticity of the film. Therefore, for a perfectly elastic material, stress and strain are in phase, *f* = 0 and the imaginary term ( *G*″) is zero. In the case of a perfectly viscous material, f = 90°, and the real part ( *G*′) is zero. The dynamic viscoelastic behavior can be more intuitively analyzed with the loss tangent (tan δ), which is equal to *E*_*v*_/ *E*_*d*_ ( Equation 6). When tan *d* > 1, *E*_*v*_ ( *G*″) is dominant, and the interface mainly exhibits viscous properties. Conversely, when tan *d* < 1, the interface mainly exhibits elastic properties, *E*_*d*_ ( *G*′), that is, if the film is purely elastic, the loss angle tangent is zero. ^32^^,^^35^^,^^38^

## 3. Results and Discussion

### 3.1 Extraction yield and physicochemical characterization

For the extraction of quinoa oil by the pressing method, it was necessary to obtain the germ of the grain by abrasive grinding as described by Roa-Acosta *et al.* ^13^ Then, it was necessary to condition the germ by heating. This is necessary to facilitate extraction due to the low granulometry of the germ flour. In addition, the effect of temperature exerts positively on the extraction yield, Adrianzén *et al*., ^39^ points out that the heat treatment of the seed facilitates the extraction process by mechanical means, because it coagulates the proteins of the cell walls, makes them permeable to the passage of the oil and also decreases the viscosity of the oil. The temperature of the resistance was 120°C, obtaining an oil with a temperature of 60°C.

3.1.1 *Moisture and volatile compounds*

Table 1 ^112^ shows the results of physical and chemical analysis of quinoa oil. It was observed that quinoa oil has 0.121% moisture and volatile matter, this low water content gives stability to the oxidation of the oil during storage, since the production of free fatty acids is slowed down. ^22^ The hydrolysis process in oils is directly proportional to the moisture content. According to Ogori, ^40^ water reacts with glycerides, producing the separation of fatty acids, monoglycerides and diglycerides, this being the reverse reaction to fat formation. On the other hand, the moisture value can be influenced according to the extraction method. Scorzza *et al.*, ^41^ found a difference when extracting almond oil by pressing and solvent extraction, with moisture and volatile matter contents of 0.17 and 5.36%, respectively. This can be attributed to the traces of solvent, making the pressing method more suitable for the food industry.

**Table 1.** Values of physicochemical parameters of quinoa oil.

Parameters	Values
Moisture and volatile matter (%)	0.12 ± 0.009
Insoluble impurities (%)	0.02 ± 0.004
Peroxide content (meq O _2_/Kg oil)	18.50 ± 0.6
Free fatty acid index (% oleic acid)	0.65 ± 0.03
Saponification index (mg KOH/g oil)	189.65 ± 0.36
Iodine value (g iodine/100 g oil)	127.36 ± 2.22
Refractive index (at 20°C)	1.47 ± 0.0003
Density (g/cm ^3^ at 20°C)	0.92 ± 0.0006

On the other hand, quinoa oil is made up of a high proportion of unsaturated fatty acids, which upon degradation generate several compounds responsible for affecting the organoleptic properties of the oil, influencing its quality. Among these compounds, the volatile ones have the lowest concentration and have the greatest effect on the aroma of the oil. ^42^^–^^44^ These compounds, mainly aldehydes and ketones, vary due to the type of seed used for vegetable oil extraction. ^45^

3.1.2 *Soluble impurities*

The content of insoluble impurities was 0.017%; low values favor oil quality and preserve shelf life, as insoluble substances deteriorate the oil. ^41^ This value is considered low compared to 0.05% in vegetable oils, which indicates that quinoa oil is mostly free of mechanical, nitrogenous, mineral impurities or soil and seed particles that can generate a bad taste, odor, or bad appearance to it. On the other hand, it also indicates that the purification method carried out by filtering and decanting was adequate to obtain a quality oil. When comparing with a quality oil such as virgin olive oil, Serrano, ^46^ found a value for this parameter between 0.047-0.099%, being in the permitted range, but higher than that found in this study. This behavior can be attributed to variables such as the type of seed used, and the processes carried out before extraction of the vegetable oil.

3.1.3 *Density*

The density for quinoa oil was 0.9179 g/cm ^3^, which is determined by the size of the fatty acids present. The number of unsaturation’s they contain, the higher the value for these, the higher the density of the oil. ^44^ Another variable to take into account for the determination is temperature, because at higher temperatures there is a dilation of the matrix leading to a decrease in density. ^39^ Quinoa oil is minimally below the density of soybean and sunflower oils. As mentioned by Alam *et al*., ^47^ the density of an oil is the product of the density of its components. The percentage present in the mixture of each one, for which variations can occur between them. Paucar *et al.,* ^48^ highlights the importance of less dense oils for cold consumption, since it is more digestible and has a lower melting point.

### 3.2 Color properties

Color constitutes a fundamental quality in sensory analysis, which can be affected by the variety and the degree of maturation of the grain, the production area, the process of obtaining and conservation. ^20^^,^^49^ The value obtained for the L* coordinate was 82 considered as a clear oil, which can reflect light. ^50^ The intense color can be affected by the coordinates a* and b*, which specify a brownish-yellow color, since b* has a high value, with yellow being the most predominant. On the other hand, the change of the a* coordinate makes it take on a slightly reddish hue. The color of quinoa oil can be influenced by the presence of compounds such as carotenoids, riboflavin and betacyanins, among others. Tang *et al.,* ^51^ states that the pigmentation of quinoa corresponds to betacyanins, being compounds that add functional value. These can be subdivided into betacyanins and betaxanthins. The first being responsible for the red-violet color and the second for the yellowish-orange color. García-Parra *et al.,* ^5^ found values for betacyanins between 0.278 and 0.883 mg/100 g and for betaxanthins between 1.1 and 13.8 mg/100 g in white quinoa grain. The coloration of the quinoa oil under study may be due to these compounds, with yellow being the most notorious when measured. However, these pigments may suffer degradation by isomerization, hydrolysis and decarboxylation when there is a thermal treatment. ^52^^–^^54^ It is pertinent to state that color is not a parameter that defines the quality of the oil, however, it turns out to be important because it exerts a strong influence on the preferences of different consumers.

### 3.3 Refractive index

Another parameter used to control the identity and quality of oils is the refractive index. It is directly related to the degree of unsaturation, confirming the content of long-chain fatty acids and is useful for observing the progress of reactions such as lipid hydrogenation. ^55^ In the quinoa oil, a refractive index of 1.4701 was observed, in accordance with the degree of unsaturation detailed in the fatty acid profile, which was 87.93%. According to Bruin, ^56^ when performing analyzes at 40°C, they found values close to those obtained in the present study, this difference can be attributed to the effect of temperature at the time of determination, since, as mentioned by Latif and Anwar, ^57^ the increase in temperature decreases the refractive index because heat destabilizes the configuration of the double bonds present in fatty acids. Similarly, Wechsler *et al.,* ^58^ reported a value of 1.4736 for sunflower oil at 20°C.

### 3.4 Peroxide index

Regarding the peroxide index, a value of 18.5 meq O _2_/Kg oil was observed. This index reflects the degree of oxidation of the oil, which is affected by the extraction conditions, since the oxidation process is initiated by the action of atmospheric oxygen and favored by temperature and exposure to light. The oil of quinoa extracted at a temperature of 120°C, which could directly affect the primary phase of peroxide formation. Gisbert *et al.,* ^59^ when comparing two extraction systems, found a higher peroxide index in the method that involves a temperature above 60°C and exposure to light. In contrast to what was reported by López-Biedma *et al.,* ^60^ the peroxide index was 14.9 meq O _2_/Kg oil, being lower than that found in the present study. It can be stated that this index in quinoa oil tends to be high due to its high degree of unsaturation, which makes it more susceptible to oxidative degradation and the formation of other compounds such as aldehydes and ketones, which are responsible for unpleasant odors and flavors characteristic of oxidized fats. ^61^ For this reason, proper handling of the flour before extraction is recommended, since, as mentioned by Ng, *et al.,* ^62^ the production of free fatty acids in quinoa flour is highly conditioned by temperature and storage time. Among other factors that influence, may be the breaking of the cells and the greater surface of contact of the flour with the oxygen during the milling process and the time elapsed between the extraction and the analysis. ^39^ The determinations were made after 24 hours while the samples completed the decantation process.

### 3.5 Free fatty acids

Regarding the index of free fatty acids, a value of 0.65% expressed in oleic acid was observed, which is within the maximum limit for natural crude oils (1%). Miranda *et al.,* ^63^ reported values between 1 and 4.1% free acidity, which are much higher compared to what was found in this study. Similarly, this depends on the type of seed, variety, state of maturation, environmental conditions where it is produced, and storage conditions of the ground seed. García-Parra *et al.*, ^5^ indicated that a high level of free acidity provides a signal regarding the state of the seed at the time of oil extraction. It should be noted that the value obtained is close to some values for different food-type oils, such as olive (0.76%) and sacha inchi (1.1%), reported by Acosta *et al.,* ^64^ and Gutiérrez *et al.,* ^65^ respectively.

This index of free fatty acids indicates low availability of water, which corroborates the previously determined moisture percentage, since it shows the low availability of water in the oil matrix that can generate hydrolysis in the triglycerides that constitute the oil, leading to the generation of free fatty acids. According to the result obtained, it can be said that the acidity index is not greatly affected by temperature compared to the peroxide index. However, a high index of free fatty acids at the time of extraction favors the oxidative deterioration of the oil. Kara *et al.,* ^66^ evaluated quality parameters of quinoa oil under storage under accelerated oxidation conditions at 60°C, where they found that there were no significant differences in terms of free acidity for 12 days. On the other hand, Xie *et al.,* ^67^ mentions that the smoke point of an oil is related to the acidity index, since lower values of this last parameter lead to raising the smoke point of the oil. ^68^

### 3.6 Saponification index

The saponification index is defined by the types of fatty acids that constitute the oil, the longer the chain, the higher the molecular weight, this means that there is a relatively smaller number of carboxylic groups. Which means that the value of the index decreases and *vice versa.* ^69^ The value found for the quinoa oil under study was 189.65 mg KOH/g oil, which, through the lipid profile, confirms the presence mainly of long-chain unsaturated fatty acids. On the other hand, Pardo *et al.,* ^70^ mentions that a high saponification index indicates the presence of a high percentage of free fatty acids. This being consistent with the results obtained, since a low presence of these was demonstrated, which allows the saponification index of quinoa oil not to exceed the established limits, considering that it has not been subjected to a process of addition of antioxidant agents that can counteract the degradation of the oil. The result was compared with sunflower and soybean oil, since these are similar in terms of fatty acid composition and for which ranges of 188-194 and 189-195 mg KOH/g oil, respectively, are established. The value of 189.65 mg KOH/g oil is within the ranges established for oils of vegetable origin intended for human consumption. Furthermore, ^71^ reported similar values for quinoa oil, 191 and 192 mg KOH/g oil, respectively.

### 3.7 Iodine index

The iodine value is an indicator of the degree of unsaturation in the oil. Values between 26 to 48 cg/100 g indicate oil saturation with a tendency to solidify, while values between 94 to 135 g I/100 g show a high level of unsaturation. ^72^ This index is used to determine the purity and identity of fats/oils; for example, the iodine values for unsaturated fatty acids, such as oleic, linoleic and linolenic acids, are 90, 181 and 274 g I/100g, respectively. ^73^ The aforementioned statement aligns with the results found in the present study, which indicate a content of 127.36 g of iodine per 100g of quinoa oil. This observation also corresponds to the fatty acid profile of quinoa oil, characterized by a notably high percentage of linoleic acid. Authors such as Caipo *et al.,* ^44^ found values between 140 and 142 g I/100 g for quinoa oil, this variation may be related to the temperature and time of heat treatment during extraction, since Adrianzén *et al*., ^39^ mentions that, by increasing these two variables, properties such as the iodine index tend to decrease due to the oxidation of the double bonds. In industry, this property is useful because it determines whether it is a drying, semi-drying, or non-drying type oil, which determines whether or not it forms films when in contact with air. The Codex Alimentarius 210-1999, ^74^ does not establish specific values for quinoa oil, however, there are limits for soybean oil from 124 to 139 g I/100 g and for sunflower from 118 to 141 g I/100 g, which were taken as a reference because they present similarity in the composition of mainly unsaturated fatty acids as mentioned above. According to the above, quinoa oil meets the parameters of these regulations for edible oils.

### 3.8 Determination of trace elements

Regarding the metallic trace elements that were determined, low values were found for nickel and copper, <0.01 mg Ni/kg and < 0.0135 mg Cu/kg, respectively, which are below the maximum limit allowed by CODEX ALIMENTARIUS 210-199, which establishes values for nickel and copper not exceeding 0.4 mg/kg. Regarding iron, the presence of 7 mg Fe/kg was determined, which is a value that exceeds the limit established by the standards, being 5 mg Fe/kg. These parameters contained in the standard are not specific to quinoa oil. However, they are taken as a reference because they are the standards that currently indicate the health requirements that must be met by oils and fats intended for human consumption. According to Gomez-Pando *et al.,* ^75^ quinoa stands out compared to cereals for its content of minerals such as copper, potassium, calcium and iron, the latter being equivalent to twice that of wheat and six times that of corn. For this reason, oils with a high content of this trace element can be obtained. Likewise, these contents depend on the type of seed, the variety, the soil where it is grown, and the chemical elements that are provided as part of its nutrition. ^76^^,^^77^ From the nutritional point of view, copper and iron are important micronutrients in human nutrition in adequate doses, fulfilling a wide range of biological functions, ^78^ among which the transport and storage of oxygen in the blood, as well as the maintenance of the nervous and immune system with the help of copper.

### 3.9 Properties bioactive

The fatty acid composition of quinoa oil obtained by gas chromatography is presented in Table 2. Next, Table 3 shows the type of fat and its content per 100 g of oil. The fatty acids present in quinoa oil are mainly unsaturated, where 59.69% were identified as polyunsaturated, with linoleic acid being the most present, while 28.24% represented monounsaturated, standing out among these, oleic acid. Saturated fatty acids represented 12.07% of the total composition. The results obtained are similar to those reported by Tang *et al.,* ^79^ for white quinoa oil, which were 12.82%, 26.82% and 60.50% for saturated, monounsaturated and polyunsaturated fatty acids, respectively. On the other hand, Tang *et al.,* ^80^ found close contents in the three solvent extraction methods, for linoleic and oleic fatty acids, being 54.80% and 24.82%, for each one, instead there was a difference to the result for linolenic acid (Alpha) between 0.49-1.82%, being lower in terms of what was obtained in the present study, which was 4%. However, Caipo *et al.,* ^44^ reported a value between 4.66-5.74% for α-linolenic acid, therefore, this value is more in agreement with the data obtained from the oil from flour of the endosperm. These results may vary slightly depending on the climate, soil and variety of the vegetable.

**Table 2.** Fatty acid profile of quinoa oil.

Fatty acid		g/100 g of sample
C 12:0	Laúric	0.02
C 14:0	Myrístic	0.17
C 14:1	Myristoleic	0.01
C 15:0	Pentadecanoic	0.06
C 16:0	Palmitic	9.91
C 16:1 cis	Palmitoleic	0.08
C 17:0	Heptadecanoic	0.05
C 18:0	Stearic	0.57
C 18:1 cis 9 Ω9	Oleic	24.9
C 18:2 cis 9,12 Ω6	Linoleic	55.29
C 18:3 cis 9,12,15 Ω3	α-Linolenic	4.00
C 20:0	Araquidic	0.44
C 20:1 cis 11 Ω9	Eicosenoic	1.65
C 20:2 cis 11, 14	Eicosadienoic	0.14
C 21:0	Heneicosanoic	0.04
C22:0	Behenic	0.58
C 22:1 cis 13 Ω9	Erucatic	1.51
C 22:2	Docosadienoic	0.10
C 23:0	Tricosanoic	0.06
C 24:0	Lignoceric	0.17
C 22:6 DHA Ω3	Docosahexanoic	0.15
C 24:1 cis 15 Ω9	Nervonic	0.09

**Table 3.** Percentage by fatty acid group in quinoa oil.

Type of fat	g/100 of sample
Saturated	12.07
Monounsaturated	28.24
Polyunsaturated	59.69
Total unsaturated	87.93
Omega 3	4.16
Omega 6	55.29
Omega 9	28.16

According to the content of fatty acids, it can be said that quinoa oil is more susceptible to oxidation, since it has a considerable percentage of fatty acids with more than one double bond in its structure (polyunsaturated). However, Paucar *et al*., ^48^ reports that oils with a higher presence of monounsaturated fatty acids, that is, with a single double bond in the chain, are less prone to oxidation, have greater stability and shelf life. In the case of oleic acid, it provides characteristics of resistance to chemical decomposition caused by high temperatures, ^39^ this is corroborated by Ceylan *et al.,* ^81^ when subjecting extra virgin olive oil, sunflower, corn and peanuts, cooked, where the most resistant oil was extra virgin olive oil, as it maintains a high concentration of oleic acid; the most susceptible was the sunflower because it had a higher content of polyunsaturated fatty acids.

From a nutritional point of view, polyunsaturated fatty acids are important, for example linoleic acid, which fulfills the function of controlling and reducing cholesterol from accumulated saturated fats, as well as being fundamental in the formation in the formation of nerve and eye tissue. α-linolenic acid allows the development of human intelligence from the fetal stage, since more than half of the fat in the brain is omega 3. ^48^ According to the National Institutes Of Health, an adult should consume 1.6 g per day; therefore, quinoa oil would supply a quarter of your requirement. However, they have disadvantages due to their instability under the effect of temperature, atmospheric oxygen and direct incidence of light, which give rise to primary oxidation initiating compounds (peroxides) ^81^ that alter the quality of the oil and lead to adverse effects on human health. Therefore, quinoa oil should avoid procedures that modify the nature of the oil or analyze its behavior in mixtures with other oils.

Lipid peroxidation, in addition to deteriorating the taste and smell of the oil, also leads to emphysema, mutagenesis, heart disease and cancer. ^79^ To counteract the effects of oxidative deterioration, various studies have been carried out to evaluate the antioxidant capacity, thus determining the bioactive compounds that can trap free radicals in food. When comparing the antioxidant capacity of the control, which was quinoa oil without additives, against another containing a concentration of BHT, it was found that the control had an induction time of 20.3 h. On the other hand, by adding BHT to quinoa oil, it was observed that the sample exceeded 21 h in its oxidative stability. However, the use of this synthetic antioxidant would not be necessary to improve this characteristic since the difference in the induction time is not considerable. Components such as phenols, carotenoids, sterols, amino acids, and oligosaccharides can improve the functional properties of vegetable oils. ^80^ Quinoa being a considerable source of vitamin E, which in addition to helping prevent lipid oxidation, is fat-soluble, which makes its presence in the oily matrix easier. Caipo ^44^ found a concentration of 1,300 ppm total tocopherols, with α-tocopherol being the most prevalent with 993 ppm. On the other hand, Abderrahim *et al.,* ^82^ found that betalains contribute to antioxidant activity. This makes quinoa oil acquire a good barrier against oxidative degradation due to rancidity. The oil showed greater oxidative stability compared to sacha inchi oil (5.1 h), soybean (17.4 h) and sunflower oil (13.1 h) added with hydroxytyrosol derivatives, this being a polyphenol with antioxidant capacities. ^83^ The results reported by Rubio (2006) for quinoa oil were 6.4 h and 8 h, which depend on the variety, these being lower than those found in the present study.

### 3.10 Rheology properties

3.10.1 *Interfacial properties and dilatational rheology*

3.10.1.1 *Adsorption kinetics*

Low molecular weight lipids with surfactant activity, such as phospholipids, glycerides, fatty acids, synthetic surfactant molecules, among others, are amphiphilic molecules that can adsorb weakly or strongly at the interface. ^84^ This behavior depends on their chemical structure, that is, their lipophilic and hydrophilic balance (HLB). On the other hand, proteins are high molecular weight biopolymers formed by peptide bonds between amino acids (monomers) that contain hydrophobic and hydrophilic residues, which allows them to adsorb at both interfaces. ^32^^,^^84^^–^^86^ Structural changes undergone by proteins may be due to the difference in molecular weight between them and lipids (minimal structural changes), and similarly, to their cooperative nature of adsorption. Therefore, protein adsorption and desorption are significantly slower than lipids at both interfaces. ^87^^,^^88^ Consequently, the presence of proteins may generate competitive adsorption at the interface. It has been reported that proteins interact with surfactant molecules in the solution differently at the surface/interface, through a hydrophobic and/or electrostatic interaction that can produce conformational changes of protein molecules in solution and at the surface/interface. ^87^

**Figure 1.**
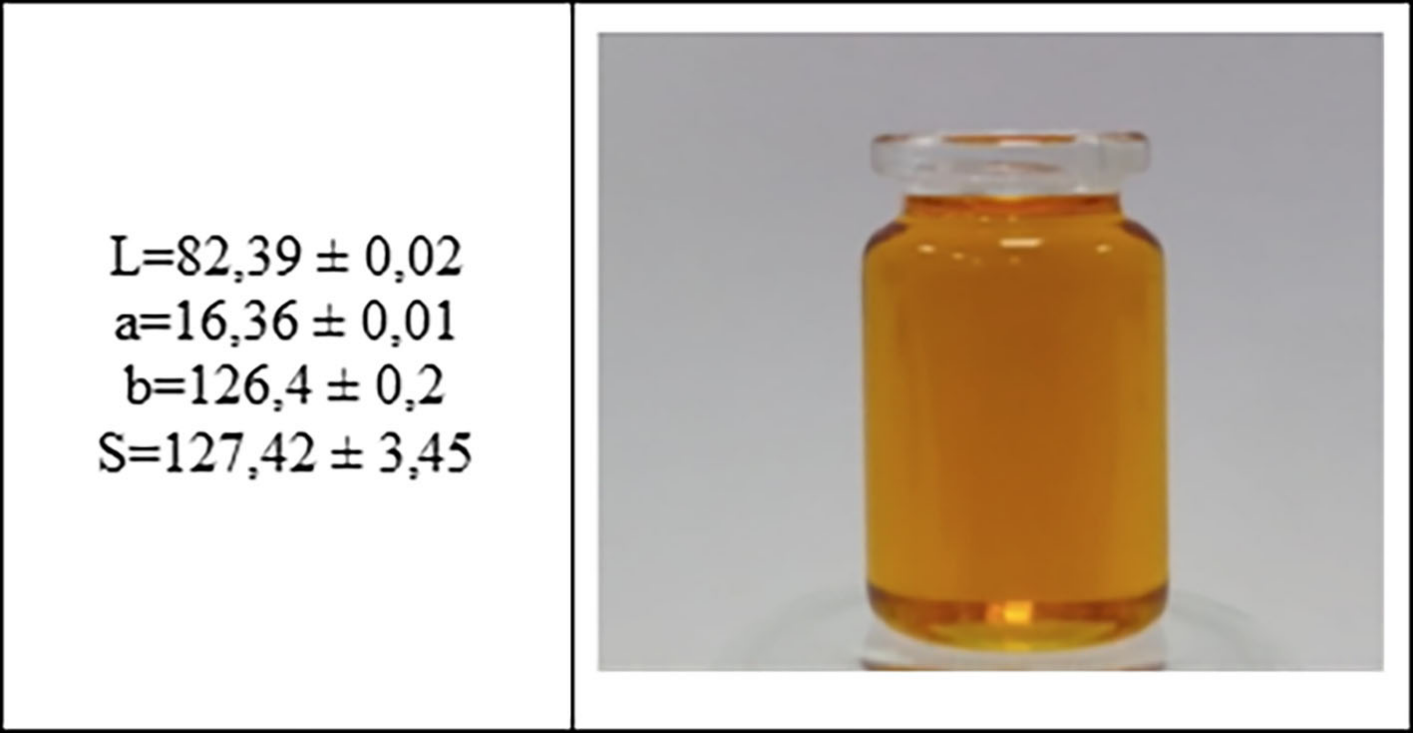
Estimated of L*, a*, b* coordinates by CIE-Lab of quinoa edible oil.

Some authors have described that oils obtained by mechanical extraction (virgin oils) contain different components, including secondary metabolites, proteins, among others. ^89^ Consequently, properties such as particle size, amorphous shape, surface roughness present in these oils, could generate surface activity. ^90^^–^^92^ Similarly, the presence of proteins in the oil contributes to the reduction of interfacial tension. ^93^^,^^94^ The adsorption dynamics of virgin oil are shown in Figure 1 at the air-oil interface. The curves obtained show a reduction in interfacial tension up to an equilibrium value of 27.1 and 27.3 mN·m ^-1^ for 10 % and 5% deformations, respectively. This decrease was small compared to equilibrium values of highly surfactant molecules such as lipids and proteins reported by other authors. ^87^^,^^88^

**Figure 2.**
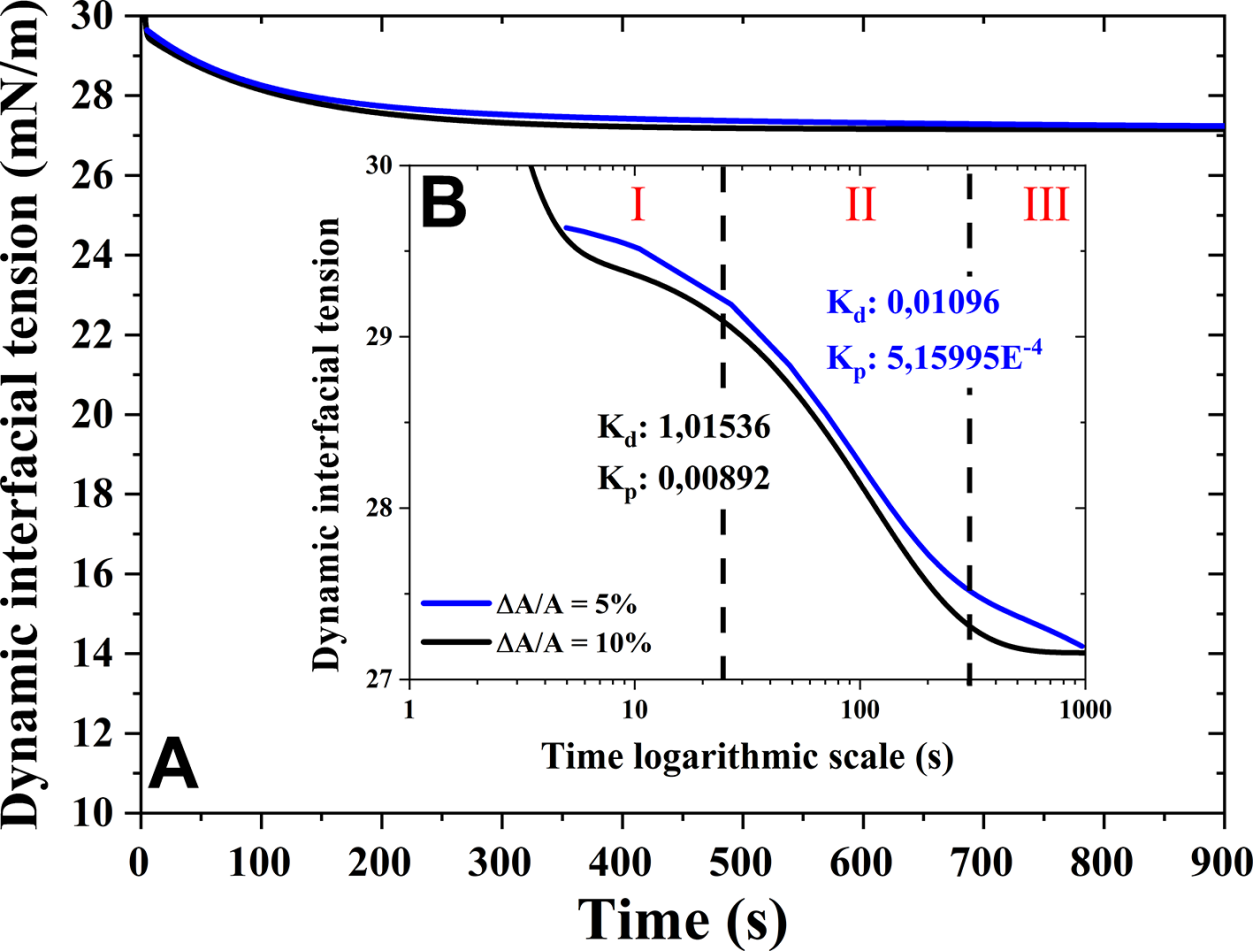
(A) Dynamic interfacial tension (γ) at the air-oil interface as a function of time for quinoa oil at room temperature. (B) The adsorption mechanisms are indicated at logarithmic time scale, as defined for individual proteins (diffusion, adsorption and penetration, and interface reorganization and equilibrium) as a function of deformation (DA/A, %).

The experimental data of the adsorption mechanisms were fitted using Equation 2. The relaxation times t _1_ and t _2_ were 85.13839 s and 477.18531 s, respectively, with a coefficient of determination (R ^2^) of 0.9996 for a deformation amplitude of 5%. Meanwhile, the relaxation times t _1_ and t _2_ for a deformation amplitude of 10% were 0.98487 s and 112.1569 s, respectively, with a coefficient of determination (R ^2^) of 0.9994. These parameters demonstrate the adsorption mechanisms during the decrease of dynamic interfacial tension. It has been observed that increasing the deformation amplitude from 5% to 10% ( Figure 2A) increases the adsorption kinetics. This could be attributed to the fact that the molecules exhibit greater molecular movement and therefore diffuse ( Figure 2B) more rapidly due to a greater fluid perturbation, causing the molecules to penetrate the interface more quickly, promoting the formation of a more stable interfacial film. Consequently, in both established deformations, t _1_ was significantly smaller than t _2_, suggesting that the adsorption of the molecules was not limited by the velocity of dynamic interfacial tension compared to the reorganization of the molecules. This means that oil molecules exhibited slow diffusion and adsorption at 5% deformations, which was not observed at 10% deformations, due to the increased molecular movement and enhanced diffusion and adsorption at the interface.

3.10.1.2 *Dilatational rheology*

**Figure 3.**
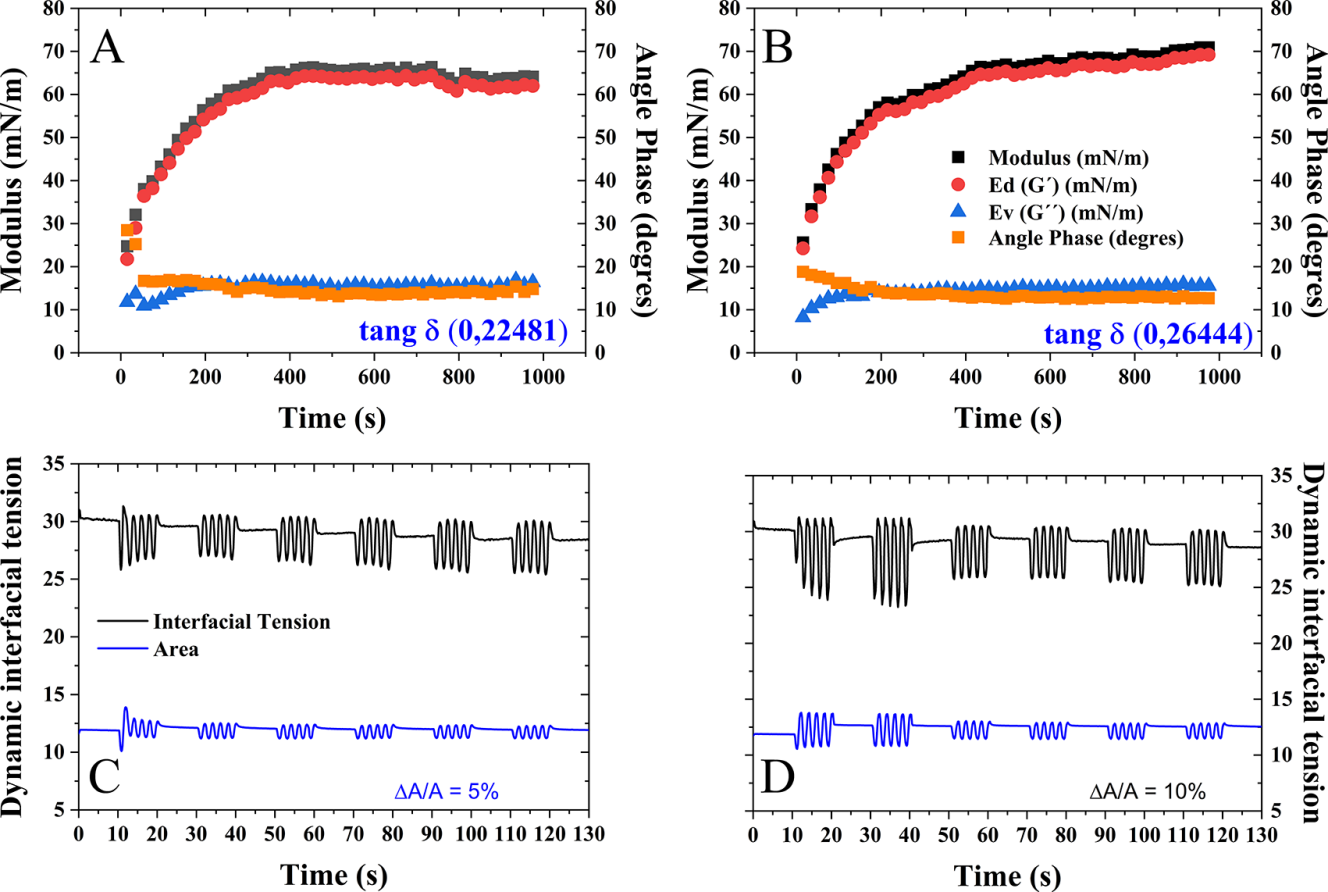
Dilational ( *E*), Elastic ( *E*_*d*_) and Viscous ( *E*_*v*_) modulus at two strain amplitudes, (A) 5% and (B) 10% with their respective sinusoidal curves of the dilational rheological behavior at different strain amplitudes (C) 5% and (D) 10%.

The analysis of the adsorption behavior of quinoa oil at the oil/water interface was performed using dilatational oscillations. Deformation sweeps experiments, analyzed in a classical manner, provide useful information only about the linear viscoelastic regime (LVR), where the viscoelastic moduli are independent of the applied deformation or stress. This response is necessary to correctly estimate *E*_*d*_ ( *G*′) and *E*_*v*_ ( *G*″) ( Figure 3A and B). Consequently, it was observed that the oscillations obtained from the interfacial tension at 5% deformations showed a smaller sinusoidal response compared to 10% deformations. Similarly, it was observed that the behavior in the tangent of delta (tan δ < 1) is less than 1, indicating a more elastic behavior (ϕ = 12), which is similar for both deformations. This behavior is ideal for the formation of emulsions with oil mixtures containing other components such as fibers (cellulose), proteins, or polysaccharides, which promote changes in structural properties within the mixtures, increasing the viscoelastic properties of this dispersed system. It is understood that when incorporating oily phases, it is necessary to stabilize them with surfactants and/or cosurfactants, depending on the industrial application. Some authors have reported that the blends between different components or binary systems can modify their sinusoidal behavior as a function of deformations. ^87^^,^^94^^–^^99^ This may be due to the different intermolecular interactions between the components that modify the structure of the dispersed system, and in turn, the linear behavior (LVR) of the dispersed system (emulsion) modifying the sinusoidal behavior of the blend. The sinusoidal curves of the dilatational rheological behavior at the different amplitudes of deformation studied, it was observed that the molecular movement of the oil molecules, at these amplitudes, respond kinetically to a thermodynamic pseudo-equilibrium from 50 s, indicating the adsorption of the molecules in a constant way that helps to form the interfacial film. It is more evident at a deformation amplitude of 10% ( Figure 3C and D). This behavior agrees with the trend shown in the dilatational modulus and the real component ( *E*_*d*_), which evidences a higher mechanical strength at higher strain amplitude.

In Figure 3A and B, a clear observation emerges: the modulus of expansion appears to be independent of the strain amplitude, and there is minimal change in the measured phase angle. These findings suggest that the adsorbed layer exhibits an inherent elastic behavior. Notably, the dilatational modulus shows an increase with rising strain amplitude. As is commonly understood, this modulus serves as a measure of resistance against the creation of surface tension gradients and the rate at which these gradients can dissipate.

Specifically, for low molecular weight surfactants, the time required for surfactant molecules to diffuse into the perturbed region or for surface molecules to reorganize and establish equilibrium decreases as the external frequency increases. Consequently, the modulus of expansion increases in response to the amplified strain amplitude.

On another note, the phase angle represents the ratio between the loss modulus and the storage modulus. Due to the shorter time of the diffusion-exchange relaxation process as the extrinsic frequency rises, the contribution of the loss modulus is reduced. This leads to a decrease in the phase angle value. Consequently, the viscous component, *E*_*v*_, decreases with increasing strain amplitude ( Figure 3B). Notably, despite these changes, the phase angle itself remains constant, indicating a higher fluidity in the interfacial film formed by the quinoa oil molecules.

To summarize, the results indicate that the adsorbed layer possesses elastic behavior, and the behavior of the dilatational modulus and phase angle is influenced by the surfactant’s diffusion-exchange relaxation process with increasing external frequency. These insights shed light on the intricate properties of interfacial films, specifically those formed by quinoa oil molecules. ^100^^,^^101^

3.10.2 *Curves flow and frequency sweep*

**Figure 4.**
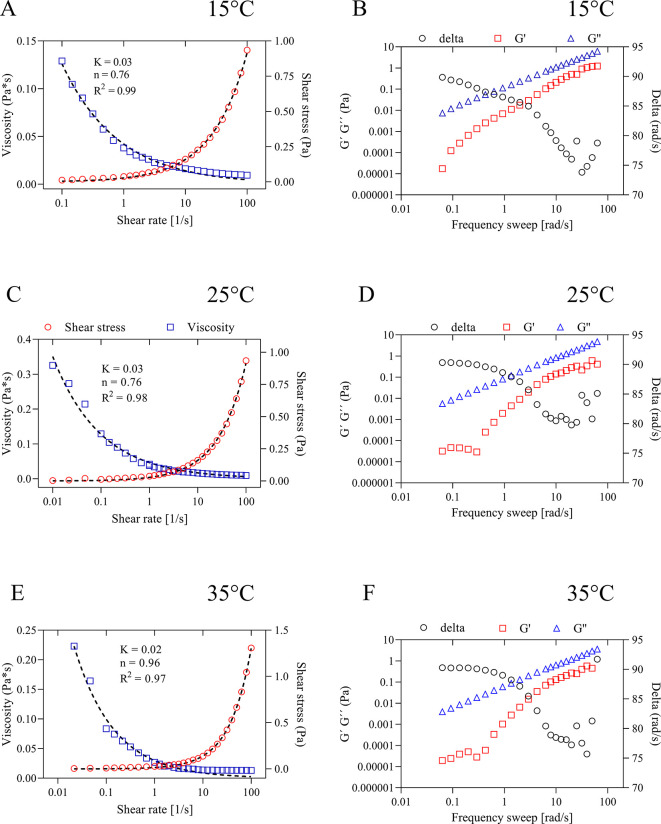
Flow curves at (A) 15°C, (C) 25°C and (E) 35°C and frequency sweeps at (B) 15°C, (D) 25°C and (F) 35°C of quinoa oil.

Figure 4 shows the flow curves ( Figure 4A, C and E) at different temperatures. It was observed that the viscosity decreases as the stress increases as a function of temperature. The flow and consistency behavior of the oil at the temperatures studied is similar, presenting a pseudoplastic behavior (n < 1) and indicating that the structure of the oil is modified as the temperature increases, presenting a shear-thinning flow.

Likewise, it was observed that the consistency index (K) decreases as the temperature increases, but the fluid index (n) of the oil increases as the shear increases. This behavior agrees with that observed in the dilatational rheological behavior of quinoa oil at 25°C. As for the frequency sweep study ( Figure 4B, D and F), it was observed that the behavior of the loss modulus ( *G*″) is similar for all the temperatures studied. However, the delta for all samples presented a value greater than 1 (tang > 1), the tendency of this result shows that quinoa oil has a viscous behavior. Similar behavior was observed in the dilatational rheology.

### 3.11 Thermal properties

The warming and cooling temperature behavior of heat flow in the fusion and crystallization process has been reported for two cycles. Two exothermic peaks were identified for crystallization ( Table 4), where the temperature values for the phase transition of the sample are between -14 and -32°C (first peak) and between -32 and -46°C (second peak). The crystallization is conditioned by the composition of fatty acids since a higher presence of polyunsaturated fatty acids (linoleic and linolenic) results in a decrease in the freezing point of the oils. ^102^

**Table 4.** Initial temperature, final temperature, and enthalpy of the different peaks of quinoa oil.

Quinoa oil	Cooling 1		Heating 1		Cooling 2		Heating 2	
	Peak 1	Peak 2	Peak 1	Peak 2	Peak 1	Peak 2	Peak 1	Peak 2
**T0 (°C)**	-15.00	-32.00	-45.12	-30.24	-14.67	-33.60	-46.47	-29.96
**TF (°C)**	-31.77	-45.31	-30.61	-8.11	-32.27	-47.18	-30.69	-8.85
**ΔH (J)**	25.91	48.33	42.43	45.98	25.78	48.77	42.91	45.56

Crystallization occurs due to the initiation of microcrystal formation (peak 1) and the freezing temperature of the sample (peak 2). ^103^ Another reason for the presence of different peaks is the cooling rate because higher cooling rates primarily exhibit exothermic behaviors, unlike lower rates where the heat flow behavior of the oil has more time to reach its thermal equilibrium, promoting its solidification with a peak of lower intensity compared to peaks at higher rates. ^104^

Similarly, triglycerides have the ability to adopt various crystalline forms, including α, β’, and β, in increasing order of stability. ^105^ This polymorphic behavior generates specific properties for each form, allowing them to be differentiated from one another. Therefore, in the crystallization process, despite having the same chemical composition, the variation in their crystalline structural configuration can also influence the presence of different exothermic peaks. Polymorphic transformations depend on different variables but mainly on the cooling rate and final temperature. ^106^

In the melting temperature, two peaks were obtained, and the starting and ending temperatures for both peaks are between -47 and -30°C (peak 1) and between -31 and -8°C (peak 2). According to Cofrades *et al*., ^107^ the first endothermic manifestation is determined by the content of tri-unsaturated fatty acids (linolenic), as the melting point decreases with increased unsaturation of the fatty acid, indicating that a higher number of double bonds implies higher energy, and therefore, no energy is required for melting. ^108^ On the other hand, Romero *et al*., ^109^ indicates that the first peak can be assigned to the fusion of one of the metastable polymorphic forms (unstable form) of a fat, and it is followed by a rearrangement of the molecules to form a new crystalline form that melts at higher temperatures. This can be correlated with the second peak (heating curve). Regarding the sum of mono-unsaturated (oleic) and di-unsaturated (linoleic) triglycerides, their composition in quinoa oil is significant since they are the most involved in the phase transformation that causes the second peak temperature, a behavior related to the appearance of a small peak due to the increase in the rate at which this process occurs. ^110^

The enthalpies calculated from the area under the curve determined on a baseline for crystallization and melting were 74,399 and 88,436 J/g, respectively, which are the sum of all transitions in the two profiles. These values are similar to those reported by Huang *et al*., ^89^ for olive oil, with a melting peak of 75 J/g, despite the DSC curves having different behaviors and the polyunsaturated/monounsaturated fat ratio being lower. Durango-Giraldo *et al*., ^111^ mention that these values are related to the chemical composition of the oil. These behaviors are related to those observed in flow curves where the consistency index and the flow index depend on the molecular ordering of the lipid molecules present in quinoa oil.

## 4. Conclusions

Quinoa oil extracted from hyperprotein flour has great potential for commercialization. It has important functional characteristics due to its content of vitamin E, betalains, carotenoids, among others. In addition, this oil showed a content of mainly unsaturated fatty acids, highlighting oleic acid (24.9%), linoleic acid (55.29%) and linolenic acid (4%), which makes it suitable for cold human consumption. Likewise, according to its physicochemical, thermal, interfacial and rheological properties characteristics, it would be useful in the formulation of new innovative products in the cosmetic industry.

## Data Availability

Zenodo: Quinoa OIL, https://doi.org/10.5281/zenodo.8165822. ^112^ This project contains the following underlying data:
- DSC.zip [thermal properties] - Interfacial Rheology.zip [interfacial properties] - Oscilltion Rheology.zip [rheological properties] - Ramcimat.zip [Oxidation Properties] Data are available under the terms of the Creative Commons Attribution 4.0 International license (CC-BY 4.0).

## References

[1] Apaza V Cáceres G Estrada R : Variedades Comerciales De Quinua En El Perú. *Organización de las Naciones Unidas para la Alimentación y la Agricultura.* 1st ed. Lima Perú: FAO;2013.

[2] Angeli V Miguel Silva P Crispim Massuela D : Quinoa (Chenopodium quinoa Willd.): An Overview of the Potentials of the “Golden Grain” and Socio-Economic and Environmental Aspects of Its Cultivation and Marketization. *Foods.* 2020 Feb 19;9(2):216. 10.3390/foods9020216 32092899 PMC7074363

[3] Garcia-Parra MÁ Roa-Acosta DF Bravo-Gomez JE : Effects of Altitudinal Gradient on Physicochemical and Rheological Potential of Quinoa Cultivars. *Front. Sustain. Food Syst.* 2022 May 16;6. 10.3389/fsufs.2022.862238

[4] Duarte B Goessling JW Fonseca VF : Quinoa variety identification based on fatty acid composition and multivariate chemometrics approaches. *J. Food Compos. Anal.* 2022 Dec;114:104798. 10.1016/j.jfca.2022.104798

[5] García-Parra M Roa-Acosta D García-Londoño V : Structural Characterization and Antioxidant Capacity of Quinoa Cultivars Using Techniques of FT-MIR and UHPLC/ESI-Orbitrap MS Spectroscopy. *Plants.* 2021 Oct 12;10(10):2159. 10.3390/plants10102159 Reference Source 34685968 PMC8539964

[6] Guerrero-López A : Impacto del cultivo de la quinua (Chenopodium quinoa Willd) como alternativa productiva y socioeconómica en la comunidad indígena Yanacona de La Vega, Cauca, Colombia. Tesis de doctorado en Agroecología. Universidad Nacional de Colombia. 2018.

[7] Steffolani ME León AE Pérez GT : Study of the physicochemical and functional characterization of quinoa and kañiwa starches. *Starch - Stärke.* 2013 Nov;65(11–12):976–983. 10.1002/star.201200286

[8] García-Parra M Roa-Acosta D Bravo-Gómez JE : Effect of the Altitude Gradient on the Physiological Performance of Quinoa in the Central Region of Colombia. *Agronomy.* 2022 Sep 5;12(9):2112. 10.3390/agronomy12092112

[9] Roa DF Buera MP Tolaba MP : Encapsulation and Stabilization of β-Carotene in Amaranth Matrices Obtained by Dry and Wet Assisted Ball Milling. *Food Bioprocess Technol.* 2017 [cited 2020 Apr 21];10(3):512–521. Reference Sources

[10] De Bock P Daelemans L Selis L : Comparison of the Chemical and Technological Characteristics of Wholemeal Flours Obtained from Amaranth ( *Amaranthus* sp.), Quinoa ( *Chenopodium quinoa*) and Buckwheat ( *Fagopyrum* sp.) Seeds. *Foods.* 2021 Mar 19;10(3):651. 10.3390/foods10030651 33808595 PMC8003493

[11] Tosi EA Ré ED Masciarelli R : Whole and Defatted Hyperproteic Amaranth Flours Tested as Wheat Flour Supplementation in Mold Breads. *LWT - Food Sci. Technol.* 2002 Aug;35(5):472–475. 10.1006/fstl.2002.0892

[12] Tosi P He J Lovegrove A : Gradients in compositions in the starchy endosperm of wheat have implications for milling and processing. *Trends Food Sci. Technol.* 2018 Dec;82:1–7. 10.1016/j.tifs.2018.09.027 30532347 PMC6267945

[13] Roa-Acosta DF Bravo-Gómez JE García-Parra MA : Hyper-protein quinoa flour (Chenopodium Quinoa Wild): Monitoring and study of structural and rheological properties. *LWT.* 2020 Dec 13 [cited 2019 Dec 14];121:108952. 10.1016/j.lwt.2019.108952 Reference Source

[14] D’Amico S Jungkunz S Balasz G : Abrasive milling of quinoa: Study on the distribution of selected nutrients and proteins within the quinoa seed kernel. *J. Cereal Sci.* 2019 Mar;86:132–138. 10.1016/j.jcs.2019.01.007

[15] Roa-Acosta DF Solanilla-Duque JF Agudelo-Laverde LM : Structural and thermal properties of the amaranth starch granule obtained by high-impact wet milling. *Int. J. Food Eng.* 2020 Oct 1 [cited 2021 Feb 13];16(10):20200024. 10.1515/ijfe-2020-0024/html

[16] Vega-Gálvez A Miranda M Vergara J : Nutrition facts and functional potential of quinoa (Chenopodium quinoa willd.), an ancient Andean grain: a review. *J. Sci. Food Agric.* 2010 Dec;90(15):2541–2547. 10.1002/jsfa.4158 20814881

[17] Cervilla N Miranda P Mufari J : Perfil de ácidos grasos en aceite de Chenopodiun quinoa Willd del Noroeste Argentino. *Conicet.* 2015;2(1):1–5.

[18] Huang R Huang K Guan X : Effect of defatting and extruding treatment on the physicochemical and storage properties of quinoa (Chenopodium quinoa Wild) flour. *LWT.* 2021 Jul;147:111612. 10.1016/j.lwt.2021.111612

[19] Benito-Román O Rodríguez-Perrino M Sanz MT : Supercritical carbon dioxide extraction of quinoa oil: Study of the influence of process parameters on the extraction yield and oil quality. *J. Supercrit. Fluids.* 2018 Sep;139:62–71. 10.1016/j.supflu.2018.05.009

[20] Wejnerowska G Ciaciuch A : Optimisation of oil extraction from quinoa seeds with supercritical carbon dioxide with co-solvents. *Czech J. Food Sci.* 2018 Feb 28;36(1):81–87. 10.17221/122/2017-CJFS

[21] Nde D Foncha A : Optimization Methods for the Extraction of Vegetable Oils: A Review. *Processes.* 2020 Feb 8;8(2):209. 10.3390/pr8020209 Reference Source

[22] Mufari JR Gorostegui HA Miranda-Villa PP : Oxidative Stability and Characterization of Quinoa Oil Extracted from Wholemeal and Germ Flours. *J. Am. Oil Chem. Soc.* 2020 Jan 25;97(1):57–66. 10.1002/aocs.12308

[23] Schiel J Hage D : Density measurements of potassium phosphate buffer from 4 to 45°C. *Talanta.* 2005 Jan 30;65(2):495–500. 10.1016/j.talanta.2004.06.029 Reference Source 18969825

[24] Gila A Bejaoui MA Beltrán G : Rapid method based on computer vision to determine the moisture and insoluble impurities content in virgin olive oils. *Food Control.* 2020 Jul;113:107210. 10.1016/j.foodcont.2020.107210 Reference Source

[25] Gutiérrez L-F Quiñones-Segura Y Sanchez-Reinoso Z : Physicochemical properties of oils extracted from γ-irradiated Sacha Inchi ( *Plukenetia volubilis* L.) seeds. *Food Chem.* 2017 Dec;237:581–587. 10.1016/j.foodchem.2017.05.148 28764039

[26] Hautfenne A : International union of pure and applied chemistry. Standard methods for the analysis of oils, fats and derivatives. *Pure Appl. Chem.* 1980;52:1939–1954.

[27] IUPAC: *International union of pure and applied chemistry, standard methods and applications.* New York, USA: Marcel Dekker;1988.

[28] Dieffenbacher A Pocklington W : Standard Methods for the Analysis of Oils, Fats and Derivatives: 1St Supplement to the 7th Revised and Enlarged Edition. Dieffenbacher A Pocklington W , editors. *IUPAC - International Union of Pure and Applied Chemistry: Applied Chemistry Division Commission on Oils, Fats and Derivatives.* 7th Editio. Boston, USA: IUPAC - International Union of Pure and Applied;1992.

[29] Baananou S Bouftira I Mahmoud A : Antiulcerogenic and antibacterial activities of Apium graveolens essential oil and extract. *Nat. Prod. Res.* 2013 Jun;27(12):1075–1083. 10.1080/14786419.2012.717284 22934666

[30] Mousavi SA Nateghi L Javanmard Dakheli M : Effects of incorporation of Chavir ultrasound and maceration extracts on the antioxidant activity and oxidative stability of ordinary virgin olive oil: identification of volatile organic compounds. *J. Food Meas. Charact.* 2022 Oct 17;16(5):4236–4250. 10.1007/s11694-022-01462-731

[31] Ortiz-Gómez V Nieto-Calvache JE Roa-Acosta DF : Preliminary Characterization of Structural and Rheological Behavior of the Quinoa Hyperprotein-Defatted Flour. *Front. Sustain. Food Syst.* 2022 May 11;6. 10.3389/fsufs.2022.852332/full

[32] Solanilla Duque JF Carrera C Patino JMR : Effect of pH on the interfacial and foaming properties of Maillard reaction-modified proteins. *Biophys. Chem.* 2022 Dec;291:106906. 10.1016/j.bpc.2022.106906 36219980

[33] Carranza-Saavedra D Váquiro HA León-Galván MF : Modelización de la adsorción en la interfase aire-agua de proteína de pescado mediante lógica borrosa Modeling adsorption at the air-water interface of fish protein by fuzzy logic. *Agron Colomb.* 2016;34((1Supl.)(March)):S362–S366.

[34] Carranza-Saavedra D Zapata-Montoya JE Váquiro-Herrera HA : Study of biological activities and physicochemical properties of Yamú (Brycon siebenthalae) viscera hydrolysates in sodium alginate-based edible coating solutions. *Int. J. Food Eng.* 2021 Sep 21;17(9):677–691. 10.1515/ijfe-2021-0036/html

[35] Tamayo Tenorio A Gieteling J Nikiforidis CV : Interfacial properties of green leaf cellulosic particles. *Food Hydrocoll.* 2017 Oct;71:8–16. 10.1016/j.foodhyd.2017.04.030

[36] Lucassen J Van Den Tempel M : Longitudinal waves on visco-elastic surfaces. *J. Colloid Interface Sci.* 1972 Dec;41(3):491–498. 10.1016/0021-9797(72)90373-6 Reference Source

[37] Lucassen J Van Den Tempel M : Dynamic measurements of dilational properties of a liquid interface. *Chem. Eng. Sci.* 1972 Jun;27(6):1283–1291. 10.1016/0009-2509(72)80104-0 Reference Source

[38] Corzo-Martínez M Carrera Sánchez C Moreno FJ : Interfacial and foaming properties of bovine β-lactoglobulin: Galactose Maillard conjugates. *Food Hydrocoll.* 2012;27(2):438–447. 10.1016/j.foodhyd.2011.11.003

[39] Adrianzén Yajahuanca N Rojas Padilla C Linares Luján G : Effect of temperature and time of thermal treatment of crushed almonds Sacha Inchi ( *Plukenetia volubilis* L.) on the performance and physical-chemical characteristics of oil obtained by cold mechanical pressing. *Agroindustrial Sci.* 2011;2:39–48.

[40] Ogori AF : Source, Extraction And Constituents Of Fats And Oils. *Food Sci. Nutr.* 2020 Apr 24;6(2):1–8. 10.24966/FSN-1076/100060

[41] Scorzza C Nieves J Vejar F : Synthesis and Physicochemical Characterization of Anionic Surfactants Derived from Cashew Nut Shell Oil. *J. Surfactant Deterg.* 2010 Jan 27;13(1):27–31. 10.1007/s11743-009-1143-5

[42] Morales MT Aparicio R : Effect of extraction conditions on sensory quality of virgin olive oil. *J. Am. Oil Chem. Soc.* 1999 Mar;76(3):295–300. 10.1007/s11746-999-0234-9

[43] Fakas S Kefalogianni I Makri A : Characterization of olive fruit microflora and its effect on olive oil volatile compounds biogenesis. *Eur. J. Lipid Sci. Technol.* 2010 Sep 13;112(9):1024–1032. 10.1002/ejlt.201000043

[44] Caipo L Sandoval A Sepúlveda B : Effect of Storage Conditions on the Quality of Arbequina Extra Virgin Olive Oil and the Impact on the Composition of Flavor-Related Compounds (Phenols and Volatiles). *Foods.* 2021 Sep 13;10(9):2161. 10.3390/foods10092161 34574270 PMC8466157

[45] Taticchi A Esposto S Veneziani G : High vacuum-assisted extraction affects virgin olive oil quality: Impact on phenolic and volatile compounds. *Food Chem.* 2021 Apr;342:128369. 10.1016/j.foodchem.2020.128369 33143966

[46] Serrano A De la Rosa R Sánchez-Ortiz A : Chemical components influencing oxidative stability and sensorial properties of extra virgin olive oil and effect of genotype and location on their expression. *LWT.* 2021 Jan;136:110257. 10.1016/j.lwt.2020.110257

[47] Alam M Sharmin E Alandis NM : Effect of organoclay on structure, morphology, thermal behavior and coating performance of Jatropha oil based polyesteramide. *e-Polymers.* 2017 Oct 26;17(6):491–500. 10.1515/epoly-2017-0096

[48] Paucar-Menacho LM Salvador-Reyes R Guillén-Sánchez J : Comparative study of physical-chemical features of sacha inchi oil (Plukenetia volubilis l.), olive oil (Olea europaea) and fish oil. *Sci. Agropecu.* 2015 Dec 31:279–290. 10.17268/sci.agropecu.2015.04.05

[49] Przygoda K Wejnerowska G : Extraction of tocopherol-enriched oils from Quinoa seeds by supercritical fluid extraction. *Ind. Crop. Prod.* 2015 Jan;63:41–47. 10.1016/j.indcrop.2014.09.038

[50] Talens C Arboleya JC Castro-Giraldez M : Effect of microwave power coupled with hot air drying on process efficiency and physico-chemical properties of a new dietary fibre ingredient obtained from orange peel. *LWT.* 2017 Apr;77:110–118. 10.1016/j.lwt.2016.11.036

[51] Tang Y Li X Zhang B : Characterisation of phenolics, betanins and antioxidant activities in seeds of three Chenopodium quinoa Willd. genotypes. *Food Chem.* 2015;166:380–388. 10.1016/j.foodchem.2014.06.018 25053071

[52] Azeredo HMC : Betalains: properties, sources, applications, and stability - a review. *Int. J. Food Sci. Technol.* 2009 Dec;44(12):2365–2376. 10.1111/j.1365-2621.2007.01668.x

[53] Abderrahim F Huanatico E Segura R : Physical features, phenolic compounds, betalains and total antioxidant capacity of coloured quinoa seeds (Chenopodium quinoa Willd.) from Peruvian Altiplano. *Food Chem.* 2015 Sep [cited 2020 Apr 28];183:83–90. 10.1016/j.foodchem.2015.03.029 25863614

[54] López-Palestina CU Aguirre-Mancilla CL Raya-Pérez JC : The effect of an edible coating with tomato oily extract on the physicochemical and antioxidant properties of garambullo (myrtillocactus geometrizans) fruits. *Agronomy.* 2018 [cited 2020 Sep 1];8(11). 10.3390/agronomy8110248 Reference Source

[55] Karabulut İ Kayahan M Yaprak S : Determination of changes in some physical and chemical properties of soybean oil during hydrogenation. *Food Chem.* 2003 Jun;81(3):453–456. 10.1016/S0308-8146(02)00397-7

[56] Bruin A De : Investigation of the Food Value of Quinua and Cañihua Seed. *J. Food Sci.* 1964 Nov;29(6):872–876. 10.1111/j.1365-2621.1964.tb00464.x

[57] Latif S Anwar F : Effect of Aqueous Enzymatic Processes on Sunflower Oil Quality. *J. Am. Oil Chem. Soc.* 2009 Apr 20;86(4):393–400. 10.1007/s11746-009-1357-8

[58] Wechsler A Zaharia M Crosky A : Macadamia (Macadamia integrifolia) shell and castor (Rícinos communis) oil based sustainable particleboard: A comparison of its properties with conventional wood based particleboard. *Mater. Des.* 2013 Sep;50:117–123. 10.1016/j.matdes.2013.03.008 Reference Source

[59] Gisbert E Andree KB Quintela JC : Olive oil bioactive compounds increase body weight, and improve gut health and integrity in gilthead sea bream (Sparus aurata). *Br. J. Nutr.* 2017 Feb 14;117(3):351–363. 10.1017/S0007114517000228 Reference Source 28245885

[60] López-Biedma A Sánchez-Quesada C Delgado-Rodríguez M : The biological activities of natural lignans from olives and virgin olive oils: A review. *J. Funct. Foods.* 2016 Oct;26:36–47. 10.1016/j.jff.2016.07.005 Reference Source

[61] Varona E Tres A Rafecas M : Methods to determine the quality of acid oils and fatty acid distillates used in animal feeding. *MethodsX.* 2021;8:101334. 10.1016/j.mex.2021.101334 Reference Source 34430240 PMC8374344

[62] Ng S-C Anderson A Coker J : Characterization of lipid oxidation products in quinoa (Chenopodium quinoa). *Food Chem.* 2007 Jan;101(1):185–192. 10.1016/j.foodchem.2006.01.016 Reference Source

[63] Miranda M Barbosa RG Trigo M : Enhancement of the rancidity stability in a marine-oil model by addition of a saponin-free quinoa (Chenopodium quinoa Willd.) ethanol extract. *Eur. J. Lipid Sci. Technol.* 2017 Sep 5 [cited 2020 Apr 28];119(9):1600291. 10.1002/ejlt.201600291

[64] Acosta-Díaz E Álvarez-Ojeda MG Guzmán-Maldonado SH : Variability of the total oil content and fatty acid profile of creole avocados from Nuevo Leon, Mexico. *Agron Mesoam.* 2019 Sep 1:705–719. 10.15517/am.v30i3.34490

[65] Gutiérrez L Sanchez-Reinoso Z Quiñones-Segura Y : Effects of Dehulling Sacha Inchi (Plukenetia volubilis L.) Seeds on the Physicochemical and Sensory Properties of Oils Extracted by Means of Cold Pressing. *J. Am. Oil Chem. Soc.* 2019 Nov 29;96(11):1187–1195. 10.1002/aocs.12270

[66] Kara K Ouanji F Lotfi EM : Biodiesel production from waste fish oil with high free fatty acid content from Moroccan fish-processing industries. *Egypt. J. Pet.* 2018 Jun;27(2):249–255. 10.1016/j.ejpe.2017.07.010

[67] Xie W Gao J Lv L : Exhaust rate for range hood at cooking temperature near the smoke point of edible oil in residential kitchen. *J. Build. Eng.* 2022 Jan;45:103545. 10.1016/j.jobe.2021.103545

[68] Afshar S Ramezan Y Hosseini S : Physical and chemical properties of oil extracted from sesame (Sesamum indicum L.) and sunflower (Helianthus annuus L.) seeds treated with cold plasma. *J. Food Meas. Charact.* 2022 Feb 26;16(1):740–752. 10.1007/s11694-021-01205-0

[69] Rocha TG Gomes PH d L Souza MCM de : Lipase Cocktail for Optimized Biodiesel Production of Free Fatty Acids from Residual Chicken Oil. *Catal. Lett.* 2021 Apr 2;151(4):1155–1166. 10.1007/s10562-020-03367-w

[70] Pardo JE Fernández E Rubio M : Characterization of grape seed oil from different grape varieties (Vitis vinifera). *Eur. J. Lipid Sci. Technol.* 2009 Feb 16;111(2):188–193. 10.1002/ejlt.200800052

[71] Ogungbenle HN : Nutritional evaluation and functional properties of quinoa (Chenopodium quinoa) flour. *Int. J. Food Sci. Nutr.* 2003 Jan 6;54(2):153–158. 10.1080/0963748031000084106 12701372

[72] Vieira B Nadaleti WC Sarto E : The effect of the addition of castor oil to residual soybean oil to obtain biodiesel in Brazil: Energy matrix diversification. *Renew. Energy.* 2021 Mar;165:657–667. 10.1016/j.renene.2020.10.056

[73] Prada F Ayala-Diaz IM Delgado W : Effect of Fruit Ripening on Content and Chemical Composition of Oil from Three Oil Palm Cultivars (Elaeis guineensis Jacq.) Grown in Colombia. *J. Agric. Food Chem.* 2011 Sep 28;59(18):10136–10142. 10.1021/jf201999d 21894914

[74] Codex Alimentarius: *Codex standard for named vegetable oils.* Codex stan.;1999; vol.210.

[75] Gomez-Pando LR Aguilar-Castellanos E Ibañez-Tremolada M : Quinoa (Chenopodium quinoa Willd.) Breeding. Al-Khayri JM Jain SM Johnson DV , editors. *Advances in Plant Breeding Strategies: Cereals.* Frist USA: Springer International Publishing;2019; p.259–316.

[76] Radovanovic V Djekic I Zarkovic B : Characteristics of Cadmium and Lead Accumulation and Transfer by Chenopodium Quinoa Will. *Sustainability.* 2020 May 7;12(9):3789. 10.3390/su12093789

[77] Koç A Çetin MD : Investigation of Some Quinoa (Chenopodium Quinoa) Genotypes in Terms of Quality Criteria. *J. Instr. Sci. Technol.* 2020 Jun 1;10:1396–1409. 10.21597/jist.631050

[78] Pehlivan E Arslan G Gode F : Determination of some inorganic metals in edible vegetable oils by inductively coupled plasma atomic emission spectroscopy (ICP-AES). *Grasas Aceites.* 2008 Sep 30;59(3). 10.3989/gya.2008.v59.i3.514

[79] Tang Y Li X Chen PX : Characterisation of fatty acid, carotenoid, tocopherol/tocotrienol compositions and antioxidant activities in seeds of three Chenopodium quinoa Willd. genotypes. *Food Chem.* 2015 May;174:502–508. 10.1016/j.foodchem.2014.11.040 25529712

[80] Tang Y Li X Chen PX : Assessing the Fatty Acid, Carotenoid, and Tocopherol Compositions of Amaranth and Quinoa Seeds Grown in Ontario and Their Overall Contribution to Nutritional Quality. *J. Agric. Food Chem.* 2016 Feb 10;64(5):1103–1110. 10.1021/acs.jafc.5b05414 26760897

[81] Ceylan MM Basturk A : Investigation of the effects of uckun (Rheum ribes L.), quinoa (Chenopodium quinoa Willd.), and propolis extracts on the thermal oxidation of palm olein oil during the deep-frying process. *J. Food Process. Preserv.* 2022 Feb 5;46(2). 10.1111/jfpp.16210

[82] Abderrahim F Huanatico E Segura R : Physical features, phenolic compounds, betalains and total antioxidant capacity of coloured quinoa seeds (Chenopodium quinoa Willd.) from Peruvian Altiplano. *Food Chem.* 2015 Sep;183:83–90. 10.1016/j.foodchem.2015.03.029 Reference Source 25863614

[83] Jiménez D Lobo M Irigaray B : Oxidative stability of baby dehydrated purees formulated with different oils and germinated grain flours of quinoa and amaranth. *LWT.* 2020 Jun;127:109229. 10.1016/j.lwt.2020.109229

[84] Rodríguez Patino JM Rodríguez Niño MR Sánchez CC : Protein–emulsifier interactions at the air–water interface. Curr Opin Colloid. *Interface Sci.* 2003 Nov;8(4–5):387–395. 10.1016/S1359-0294(03)00095-5 Reference Source

[85] Perez AA Carrara CR Sánchez CC : Interfacial dynamic properties of whey protein concentrate/polysaccharide mixtures at neutral pH. *Food Hydrocoll.* 2009 Jul;23(5):1253–1262. 10.1016/j.foodhyd.2008.08.013 Reference Source

[86] Dickinson E : Hydrocolloids at interfaces and the influence on the properties of dispersed systems. *Food Hydrocoll.* 2003;17(1):25–39. 10.1016/S0268-005X(01)00120-5

[87] McClements DJ : *Food Emulsions: Principles, Practices, and Techniques.* McClements DJ , editor. third USA: CRC Press;2015;714p. Reference Source

[88] Maget-Dana R : The monolayer technique: a potent tool for studying the interfacial properties of antimicrobial and membrane-lytic peptides and their interactions with lipid membranes. *Biochim. Biophys. Acta Biomembr.* 1999 Dec;1462(1–2):109–140. 10.1016/S0005-2736(99)00203-5 Reference Source 10590305

[89] Huang K Zhong P Xu B : Discrimination on potential adulteration of extra virgin olive oils consumed in China by differential scanning calorimeter combined with dimensionality reduction classification techniques. *Food Chem.* 2023 Mar;405:134996. 10.1016/j.foodchem.2022.134996 Reference Source 36435104

[90] Deshmukh OS Ende D van den Stuart MC : Hard and soft colloids at fluid interfaces: Adsorption, interactions, assembly &amp; rheology. *Adv. Colloid Interf. Sci.* 2015 Aug;222:215–227. 10.1016/j.cis.2014.09.003 Reference Source 25288385

[91] Li Z Harbottle D Pensini E : Fundamental Study of Emulsions Stabilized by Soft and Rigid Particles. *Langmuir.* 2015 Jun 16;31(23):6282–6288. 10.1021/acs.langmuir.5b00039 25835257

[92] Madivala B Vandebril S Fransaer J : Exploiting particle shape in solid stabilized emulsions. *Soft Matter.* 2009;5(8):1717. 10.1039/b816680c Reference Source

[93] Evans M Ratcliffe I Williams PA : Emulsion stabilisation using polysaccharide–protein complexes. *Curr. Opin. Colloid Interface Sci.* 2013 Aug;18(4):272–282. 10.1016/j.cocis.2013.04.004 Reference Source

[94] Randall RC Phillips GO Williams PA : The role of the proteinaceous component on the emulsifying properties of gum arabic. *Food Hydrocoll.* 1988 Jun;2(2):131–140. 10.1016/S0268-005X(88)80011-0 Reference Source

[95] Tcholakova S Denkov ND Lips A : Comparison of solid particles, globular proteins and surfactants as emulsifiers. *Phys. Chem. Chem. Phys.* 2008;10(12):1608–1627. 10.1039/b715933c Reference Source 18338062

[96] Marefati A Wiege B Abdul Hadi N : In vitro intestinal lipolysis of emulsions based on starch granule Pickering stabilization. *Food Hydrocoll.* 2019;95(December 2018):468–475. 10.1016/j.foodhyd.2019.04.051

[97] Xu X Luo L Liu C : Influence of electrostatic interactions on behavior of mixed rice glutelin and alginate systems: pH and ionic strength effects. *Food Hydrocoll.* 2017 Feb;63:301–308. 10.1016/j.foodhyd.2016.09.005 Reference Source

[98] McClements DJ Decker E : Interfacial Antioxidants: A Review of Natural and Synthetic Emulsifiers and Coemulsifiers That Can Inhibit Lipid Oxidation. *J. Agric. Food Chem.* 2018;66(1):20–35. 10.1021/acs.jafc.7b05066 29227097

[99] Felix M Yang J Guerrero A : Effect of cinnamaldehyde on interfacial rheological properties of proteins adsorbed at O/W interfaces. *Food Hydrocoll.* 2019;97(May):105235. 10.1016/j.foodhyd.2019.105235

[100] López-Castejón ML Bengoechea C Díaz-Franco J : Interfacial and Emulsifying Properties of Quinoa Protein Concentrates. *Food Biophys.* 2019;15:122–132. 10.1007/s11483-019-09603-0

[101] Rodríguez Patino JM Navarro García JM Rodríguez Niño MR : Protein–lipid interactions at the oil–water interface. *Colloids Surfaces B Biointerfaces.* 2001 Jul;21(1–3):207–216. 10.1016/S0927-7765(01)00173-4 11377949

[102] Pérez-Jiménez F Ruano J Perez-Martinez P : The influence of olive oil on human health: not a question of fat alone. *Mol. Nutr. Food Res.* 2007 Sep 19. 10.1002/mnfr.200600273 17879991

[103] Drinić Z Mudrić J Zdunić G : Effect of pomegranate peel extract on the oxidative stability of pomegranate seed oil. *Food Chem.* 2020 Dec;333:127501. 10.1016/j.foodchem.2020.127501 Reference Source 32682230

[104] Bañares C Martin D Reglero G : Protective effect of hydroxytyrosol and rosemary extract in a comparative study of the oxidative stability of Echium oil. *Food Chem.* 2019 Aug;290:316–323. 10.1016/j.foodchem.2019.03.141 Reference Source 31000052

[105] Haque Akanda MJ Norazlina MR Azzatul FS : Hard Fats Improve the Physicochemical and Thermal Properties of Seed Fats for Applications in Confectionery Products. *Food Rev. Int.* 2020 Aug 17;36(6):601–625. 10.1080/87559129.2019.1657443

[106] Li MF He ZY Li GY : The formation and characterization of antioxidant pickering emulsions: Effect of the interactions between gliadin and chitosan. *Food Hydrocoll.* 2019;90(September 2018):482–489. 10.1016/j.foodhyd.2018.12.052

[107] Cofrades S Antoniou I Solas MT : Preparation and impact of multiple (water-in-oil-in-water) emulsions in meat systems. *Food Chem.* 2013;141(1):338–346. 10.1016/j.foodchem.2013.02.097 23768366

[108] Bonilla-Loaiza AM Váquiro-Herrera HA Solanilla-Duque JF : Physicochemical and bioactive properties of avocado (Persea americana Mill. cv. Lorena). *Int. J. Food Eng.* 2022 Apr 22;18(4):303–315. 10.1515/ijfe-2021-0237/html

[109] Romero S Minari RJ Collins SE : Bio-paraffin from Soybean Oil as Eco-friendly Alternative to Mineral Waxes. *Ind. Eng. Chem. Res.* 2021 Apr 21;60(15):5364–5373. 10.1021/acs.iecr.1c00322

[110] Gila A Sánchez-Ortiz A Jiménez A : The ultrasound application does not affect to the thermal properties and chemical composition of virgin olive oils. *Ultrason. Sonochem.* 2021 Jan;70:105320. 10.1016/j.ultsonch.2020.105320 Reference Source 32890985 PMC7786558

[111] Durango-Giraldo G Zapata-Hernandez C Santa JF : Palm oil as a biolubricant: Literature review of processing parameters and tribological performance. *J. Ind. Eng. Chem.* 2022 Mar;107:31–44. 10.1016/j.jiec.2021.12.018 Reference Source

[112] Solanilla Duque JF : Quinoa OIL.[Dataset]. *Zenodo.* 2023. 10.5281/zenodo.8165822

