# Supplementary material for: Preliminary study of physicochemical, thermal, rheological, and interfacial properties of quinoa oil
Source: F1000Res. 2023 Nov 15;12:1477. [Version 1] doi: 10.12688/f1000research.134134.1 (PMC11162528; doi:10.12688/f1000research.134134.1)
Supplement: Peer review history

# Reviewer report: Reviewer response for version 1

**DOI:** 10.5256/f1000research.147162.r261200

**Published:** 2024-06-08

**Authors:** Hamdy A Zahran

**Affiliations:** 1 Fats and Oils Dept., Food Industries and Nutrition Research Institute, National Research Centre, Dokki, Egypt

Dear Author,

 - My comments were mentioned on the attached PDF file found here

 - sufficient details of methods and analysis are required especially for oil content calculation for your matrix

 -  the conclusions aren't adequately supported by the results, it needs some revision 

 - some results for oxidative stability are concerning 

 Regards

Is the work clearly and accurately presented and does it cite the current literature?

Yes

If applicable, is the statistical analysis and its interpretation appropriate?

Yes

Are all the source data underlying the results available to ensure full reproducibility?

Yes

Is the study design appropriate and is the work technically sound?

Yes

Are the conclusions drawn adequately supported by the results?

Partly

Are sufficient details of methods and analysis provided to allow replication by others?

No

Reviewer Expertise:

Fats and oils chemistry and technology

I confirm that I have read this submission and believe that I have an appropriate level of expertise to confirm that it is of an acceptable scientific standard, however I have significant reservations, as outlined above.

# Reviewer report: Reviewer response for version 1

**DOI:** 10.5256/f1000research.147162.r225966

**Published:** 2024-05-11

**Authors:** Anuchita Moongngarm

**Affiliations:** 1 Department of Food Technology and Nutrition, Faculty of Technology, Mahasarakham University, Maha Sarakham, Maha Sarakham, Thailand

The manuscript (MS) is well-written and organized. It contains some interesting information. However, to improve the MS quality to meet the standard of the journal, a revision is required.

 1. The title may be revised; "Preliminary study of" may be deleted.

 2. Introduction section, the second paragraph should be rewritten to make it clear and relevant to the justification. The third paragraph of the introduction should be improved to indicate the rationale and knowledge gap that the authors need to investigate. The information about the advantages of physical oil extraction in previous studies should be added, more literature review is required.

 2. Methods and results

 Authors should also conduct or provide the results of oil extraction using solvent or conventional methods to compare with the proposed method in this study. The results reported in this version didn't compare with any treatments making the research less interesting.

Is the work clearly and accurately presented and does it cite the current literature?

Yes

If applicable, is the statistical analysis and its interpretation appropriate?

Yes

Are all the source data underlying the results available to ensure full reproducibility?

Yes

Is the study design appropriate and is the work technically sound?

Partly

Are the conclusions drawn adequately supported by the results?

Yes

Are sufficient details of methods and analysis provided to allow replication by others?

Yes

Reviewer Expertise:

Food chemistry, Food analysis, Functional foods

I confirm that I have read this submission and believe that I have an appropriate level of expertise to confirm that it is of an acceptable scientific standard, however I have significant reservations, as outlined above.

# Reviewer report: Reviewer response for version 1

**DOI:** 10.5256/f1000research.147162.r234748

**Published:** 2024-02-01

**Authors:** Edward Rój

**Affiliations:** 1 Łukasiewicz Research Network-New Chemical Syntheses Institute, Puławy, Poland

**Review of the publication "Preliminary study of physicochemical, thermal, rheological, and interfacial properties of quinoa oil**

 General information:

 The authors performed a very large scope of work determining the physicochemical properties of quinoa oil, moisture and volatile oil content, impurity content, oil color, physical and chemical quality indexes, bioactive properties, thermal and rheological properties, interfacial properties and dilatational rheology.

**Notes details**

 Is the work clearly and accurately presented and does it cite the current literature?

 • The work concerns preliminary research on quinoa seed endosperm oil produced mechanically using an automatic auger extractor. The scope of the research was wide, including preparation of the raw material for extraction, oil extraction and determination of physicochemical properties, including density, humidity, volatile oils, impurities, color properties, determination of physical and chemical quality indexes, peroxide index, free fatty acis index, saponification index, iodine index, metallic trace elements, bioactive properties, thermal properties, rheological properties, interfacial properties and dilatational rheology. The authors cited 112 references. The literature is well selected thematically and is up-to-date. There are even a few entries from 2023.

 Is the study design appropriate and does the work have academic merit?

 • The study design is designed correctly. Due to the detailed descriptions of research methods and materials used, the paper has great academic value both in terms of research, teaching and practice. The presented research methods can be used by both students and employees of research laboratories.

 Are sufficient details of methods and analysis provided to allow replication by others?

 • All presented methods are developed in detail and can be repeated. If the authors used other methods that were not developed by them, information about them was provided in the cited literature.

 If applicable, is the statistical analysis and its interpretation appropriate?

 • The values of the physicochemical parameters of quinoa oil are given after statistical processing. Mean values and standard deviations are provided. Fatty acid profile of quinoa oil contains average data. They were probably made in only one repetition. The oil color parameters were statistically processed and include average values and standard deviations. Rheological tests were carried out meticulously and statistical analysis of the results was carried out. Thermal properties tests were performed in one repetition, probably due to the high time consumption.

 Are all the source data underlying the results available to ensure full reproducibility?

 • The authors have posted files with source data at the indicated e-mail address. The address is active and the reviewer has checked access.

 Are the conclusions drawn adequately supported by the results?

 • Individual tasks and the obtained results are extensively discussed in the Results and Discussion chapter. The results were compared with those of the cited authors. The conclusions are modest and constitute a short summary of the results obtained.

 • Research in the field of the interfacial and rheological properties is original in relation to consumer oils. The information provided is of great value to the materials industry for the stabilization of emulsions, gels, and foams and other materials.

**Notes to Authors:**

 • Due to the high content of unsaturated fatty acids in the oil, the proposed production technology does not seem to be the best solution due to the relatively high extrusion temperature (120 ^0^C) and the possibility of oil contact with air. A better solution would be to use another technology, e.g. supercritical CO2 extraction, which is unfortunately a more expensive solution.

 • Moreover, the proposed pressing technology is suitable for raw materials with fatty acid content above 15%. However, there is quite a lot of oil left in the cake, often at the level of 5-8%. To improve efficiency, high pressing temperatures of up to 200 ^0^C are often used, which can significantly deteriorate the quality of the oil and residue.

 • In the tested raw material, the acid ratio (omega 3):(omega 6) is 4.16:55.29 (see table 3), which corresponds to approximately 1/13. Meanwhile, for consumption purposes, the optimal ratio of (omega 3): (omega 6) for consumers is considered to be 1:1 to 1:5 (see Trends in Food Science & Technology 120 (2022) 140–153). Therefore, for consumption purposes, for health safety reasons, this oil should be blended with other oils to obtain the desired or similar omega 3/omega 6 composition.

 • The conclusions stated that the oils additionally contain carotenoids, betalains and vitamin E, but these are only minor mentions. The same applies to the mention of the uses of oil in the cosmetics industry. This information should not be included in the summary because it was not the subject of broader research and was not discussed in the text of the publication. But they could possibly be included in the chapter on expected development trends in research on other biologically active compounds in quinoa oil.

Is the work clearly and accurately presented and does it cite the current literature?

Yes

If applicable, is the statistical analysis and its interpretation appropriate?

Yes

Are all the source data underlying the results available to ensure full reproducibility?

Yes

Is the study design appropriate and is the work technically sound?

Yes

Are the conclusions drawn adequately supported by the results?

Partly

Are sufficient details of methods and analysis provided to allow replication by others?

Yes

Reviewer Expertise:

Supercritical extraction technology, bioactive properties of natural extracts, process optimisation, analytical methods.

I confirm that I have read this submission and believe that I have an appropriate level of expertise to confirm that it is of an acceptable scientific standard.

## References

[1] : Futuristic food fortification with a balanced ratio of dietary ω-3/ω-6 omega fatty acids for the prevention of lifestyle diseases. *Trends in Food Science & Technology* .2022;120: 10.1016/j.tifs.2022.01.006 140-153 10.1016/j.tifs.2022.01.006
